# Average interradicular sites for miniscrew insertion: should dental crowding be considered?

**DOI:** 10.1590/2177-6709.22.5.090-097.oar

**Published:** 2017

**Authors:** Michele Tepedino, Paolo M. Cattaneo, Francesco Masedu, Claudio Chimenti

**Affiliations:** 1 University of L’Aquila, Department of Biotechnological and Applied Clinical Sciences (L’Aquila, Italy).; 2 Aarhus University, Faculty of Health, Department of Dentistry, Section of Orthodontics (Aarhus, Denmark).

**Keywords:** Orthodontic mini-implant, Digital models, Anatomy, Safe zones

## Abstract

**Objective:**

To define a map of interradicular spaces where miniscrew can be likely placed at a level covered by attached gingiva, and to assess if a correlation between crowding and availability of space exists.

**Methods:**

Panoramic radiographs and digital models of 40 patients were selected according to the inclusion criteria. Interradicular spaces were measured on panoramic radiographs, while tooth size-arch length discrepancy was assessed on digital models. Statistical analysis was performed to evaluate if interradicular spaces are influenced by the presence of crowding.

**Results:**

In the mandible, the most convenient sites for miniscrew insertion were in the spaces comprised between second molars and first premolars; in the maxilla, between first molars and second premolars as well as between canines and lateral incisors and between the two central incisors. The interradicular spaces between the maxillary canines and lateral incisors, and between mandibular first and second premolars revealed to be influenced by the presence of dental crowding.

**Conclusions:**

The average interradicular sites map hereby proposed can be used as a general guide for miniscrew insertion at the very beginning of orthodontic treatment planning. Then, the clinician should consider the amount of crowding: if this is large, the actual interradicular space in some areas might be significantly different from what reported on average. Individualized radiographs for every patient are still recommended.

## RESUMO

**Objetivo:**

elaborar um mapa dos espaços inter-radiculares nos quais os mini-implantes podem ser inseridos, em um nível coberto por gengiva inserida; e avaliar se existe correlação entre o apinhamento dentário e a disponibilidade de espaços.

**Métodos:**

radiografias panorâmicas e modelos digitais de 40 pacientes foram selecionados seguindo critérios de inclusão. Os espaços inter-radiculares foram medidos nas radiografias panorâmicas, enquanto a discrepância de modelo foi avaliada nos modelos digitais. Realizou-se, então, uma análise estatística para avaliar se os espaços inter-radiculares foram influenciados pela presença do apinhamento dentário.

**Resultados:**

na mandíbula, os locais mais adequados para a inserção dos mini-implantes foram os espaços compreendidos entre os segundos molares e primeiros pré-molares; na maxila, entre os primeiros molares e segundos pré-molares, bem como entre caninos e incisivos laterais, e entre os dois incisivos centrais. Os espaços inter-radiculares entre os caninos e incisivos laterais superiores e entre o primeiro e o segundo pré-molares inferiores mostraram-se influenciados pela presença do apinhamento dentário.

**Conclusões:**

o mapa dos espaços inter-radiculares mais adequados aqui proposto pode ser adotado como um guia geral para a inserção de mini-implantes, e pode ser usado logo ao início do planejamento do tratamento ortodôntico. Em seguida, o clínico deve levar em consideração a quantidade de apinhamento: caso esse seja grande, o real espaço inter-radicular, em algumas áreas, poderá ser significativamente diferente da média aqui relatada. Assim, recomenda-se que sempre sejam feitas radiografias individualizadas para cada paciente.

## INTRODUCTION

Orthodontic miniscrews are devices specifically designed to be temporarily inserted in the maxillofacial bones to provide anchorage for an orthodontic appliance.^1^ They are commonly used when patient compliance is an issue, when there are insufficient teeth to assure an appropriate biomechanics, or when anchorage management is critical.^2^ Their success rate is reported to be between 61% and 100%,^3^ ^-^ ^5^ and is affected by many factors: miniscrews dimensions, geometry, surface characteristics, surgical technique and clinician’s experience, bone quantity and quality, loading force, primary stability and oral hygiene.^3^ ^,^ ^6^ ^-^ ^9^ Also, root proximity appears to also have a role.^10^ Therefore, the choice of an appropriate insertion site is critical.

Many authors have tried to define a map of “safe zones” for miniscrews insertion: Schnelle et al^11^ assessed on panoramic radiographs the presence of at least 3 or 4 mm of bone between two adjacent roots, in order to define a map of the interradicular sites where most likely a miniscrew can be safely placed. The authors decided to use the reference of 3 and 4 mm of space considering a miniscrew diameter between 1.2 and 2 mm, and the need of at least 1 mm of bone around the miniscrew. Other authors measured the space between roots at different levels on panoramic radiographs^12^ or Cone Beam Computed Tomography (CBCT).^13^ ^-^ ^17^ Nevertheless, in order to have a convenient primary stability of the miniscrew, the quality of the cortical bone seems to be crucial; thus, many authors have investigated the variation of thickness of the buccal cortical plate of maxillary and mandibular bone between different sites.^14^ ^,^ ^15^ ^,^ ^18^ ^,^ ^19^ One study used micro-CTs on autopsy material to evaluate the cortical thickness in the posterior region of maxillary and mandibular bones and the possible interference with the maxillary sinus.^20^ From a systematic literature review emerges that the ideal sites for the placement of orthodontic miniscrew in both the maxilla and the mandible, taking into consideration quantity and quality of bone, are the buccal and lingual interradicular spaces between the second premolar and the second molar.^21^ Moreover, another important aspect for the success of miniscrews insertion is their placement in the attached gingiva:^22^ ^,^ ^23^ indeed, this is not affected by tissue movements, hygiene maneuvers are simpler and the risk of tissue irritation is lower. Some authors^11^ ^,^ ^16^ measured the interradicular spaces together with the height of attached gingiva. These investigations revealed that, in many cases, the requested amount of interradicular bone is first available far beyond the muco-gingival line. 

An interesting information is that the availability of bone between the roots is influenced by the position of the teeth, for example when a malocclusion or dental crowding is present. A study showed that for different malocclusions there are differences in bone availability between the roots;^24^ the authors related this finding to changes in teeth axial inclination depending on dentoalveolar compensation, which is a consequence of the presence of a skeletal malocclusion. Also, Schnelle et al^11^ found that in orthodontic patients, after tooth alignment there were more available spaces for miniscrews positioning than before treatment. To the knowledge of the authors, no one tried to find if there is a correlation between quantity of interradicular bone and dental crowding.

The objectives of this study were: 1) to define an average map of the interradicular spaces where is possible to find at least 3 mm of bone available for miniscrew insertion at an height level that is likely to be covered by attached gingiva; 2) to investigate whether it is possible to estimate the interradicular bone availability for miniscrew positioning considering the crowding of the arches, in order to give to the clinician a possible non-radiological method to estimate the likelihood of miniscrew insertion. The null hypothesis was that no correlation exists between dental crowding and amount of interradicular space.

## MATERIAL AND METHODS

No previous work assessed interaction between dental crowding and interradicular spaces; therefore, it was not possible to retrieve suggestions from the literature about a plausible correlation coefficient r estimate. According to Cohen’s criteria,^25^ an expected r* *= 0.50 corresponding to a large effect size was assumed: having a power of 90% and a Type I error of 5%, a sample size estimate of n = 38 was calculated.

The records of orthodontic patients consecutively treated at the orthodontic department of the University of L’Aquila from January 2012 to December 2013 were screened. Sixty-two patients were selected according to the following inclusion criteria:

» Age between 14 and 35 years.
» Full permanent dentition, with all the second molars fully erupted.
» No agenesis or missing teeth.
» Absence of signs of periodontal disease assessed on panoramic radiographs.

For each patient, pre-treatment digital panoramic radiograph and dental casts were collected. The operator who made the measurements was blinded so that it was not possible to associate one panoramic radiograph with the respective plaster model. This was done by assigning a random number to each panoramic radiograph and dental cast, using an online tool (www.randomizer.org). 

### Panoramic radiographs

All the radiographs were taken by the same, well-trained dental radiologist, with the same machine (Orthopantomograph^®^ OP200, Instrumentarium Dental, Tuusula, Finland) on a consecutive period of time. All the measurements were carried out by the same operator using Adobe Photoshop CS3 software (Adobe Systems Incorporated, San Jose, California, USA).

**Figure 1:**
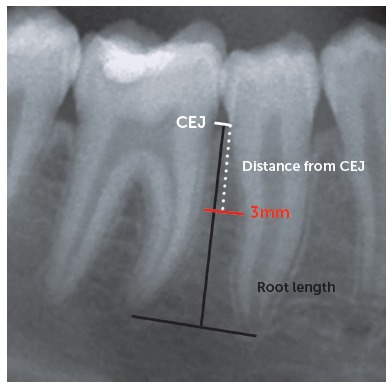
Schematic representation of the method used to measure interradicular spaces. First, the cemento-enamel junction (CEJ) of the two adjacent teeth was identified. Starting from the CEJ, a ruler was scrolled down toward the root apex until 3 mm of horizontal interradicular space was found; then, the distance from the CEJ was measured as well as the total root length from the CEJ to the apex.

Starting from the interradicular space mesial to the second molar, a virtual ruler was moved down perpendicular to the roots starting from the cemento-enamel junction (CEJ) toward the root apex, until 3 mm of space between the roots of the two adjacent teeth were found (Fig 1). The vertical distance from the CEJ to this point was recorded: to avoid the measurement error caused by the vertical magnification present in the radiographs, this distance was used to calculate a ratio based on the shorter length of the two roots adjacent to the actual interradicular space. In this way, the distance could be expressed as a percentage of the roots length, instead of a distance in millimeters. A reported value of 100% therefore means that a space of at least 3 mm was not available between the two considered adjacent roots at any distance from the CEJ. The horizontal measurements of 3 mm of interradicular space were adjusted accordingly to the average magnification factor reported by the manufacturer of the radiographic machine.

These measurements were then used to define a map of interradicular spaces for miniscrew insertion. The interradicular spaces where 3 mm of horizontal space were found at 50% or less of the root length were considered ideal insertion sites. If the 3 mm were present further toward the apex, the interradicular spaces were considered borderline (from 51% to 75% of the root length) or unfavorable sites (beyond 76%).

### Digital dental models

To measure the crowding of the arches, the dental casts were digitized using an optic scanner (D250, 3Shape, Copenhagen, Denmark) and saved in the STL (stereolithographic) format using a dedicated software (ScanIt Orthodontics 2013, v. 5.5.1.3, 3Shape, Copenhagen, Denmark). The models were thus imported into a model analyzer software (OrthoAnalyzer 2013, v. 1.5.1.3, 3Shape, Copenhagen, Denmark) where arch crowding was assessed. 

The crowding of the arches was measured by determining the arch-tooth size discrepancy: the width of the teeth, from second premolar to second premolar, was measured, then the length of the alveolar base, from the mesial contact point of the first molars, was determined by mean of a curved line passing through the contact point of each teeth.^26^

Crowding values, expressed in millimeters, were obtained from the whole maxillary and mandibular arches, for the anterior segments (between right and left canine) and for the two posterior segments (mesial to the first molar and distal to the canine) in both arches.

### Error of the method

To measure the error of the method, the same operator repeated all the measurements concerning interradicular spaces and crowding after one week, over 20 randomly selected panoramic radiographs and 20 digital models, and the results were compared using the Dahlberg’s formula to assess the presence of random errors, and paired samples *t*-test, to account for any systematic error (*p*< 0.05).

### Statistical analysis

To assess data distribution, the one-sample Kolmogorov-Smirnov test was performed for all the variables. The independent sample *t*-test or the Mann-Whitney U test, depending if data were normally distributed or not, was performed to compare all the data of the left and the right sides; if no statistically significant difference was found, the data from left and right side were pooled. To evaluate a possible correlation between crowding and availability of interradicular bone, a Pearson correlation or a Kendall’s tau b test, depending on data distribution, was performed. For all the tests, the significance level was set at 0.05.

## RESULTS

Of the 62 selected patients, 22 were excluded because of non-adequate quality of radiographs and/or plaster models; therefore, only 40 patients were included in the study sample.

The Dahlberg formula revealed a good reliability for the measurements of interradicular spaces (1.9 ± 1.6%, range 0.1 - 5.4%) and for the assessment of crowding on digital models (0.23 ± 0.04 mm, range 0.18 - 0.27 mm). Paired samples *t*-test revealed no systematic errors, since all the comparisons were not statistically significant.

In the maxilla, all data concerning interradicular space were not normally distributed, except for the measurements of the interradicular space between canine and lateral incisor of both sides, as well as between the maxillary central incisors, which were normally distributed. In the mandible, all the data were normally distributed except for those concerning the spaces between all the four mandibular incisors. Since all the data regarding crowding were not normally distributed, non-parametric tests were used. The Mann-Whitney U test between data from left and right side revealed no statistically significant difference, therefore data for all interradicular spaces as well as for crowding of the posterior segments were pooled.

**Figure 2:**
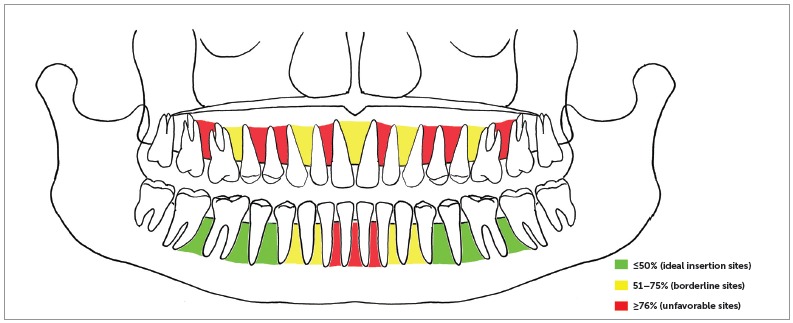
Map of interradicular spaces measured on panoramic radiographs. Values are expressed as a ratio between the total root length and the distance of the 3 mm of space from the CEJ.

In the maxilla, for all the interradicular spaces, 3 mm of available bone on average were present far beyond halfway the length of the roots (Table 1). The lowest values were found between first molar and second premolar, between canine and lateral incisor and between the two central incisors. In the mandible 3 mm of interradicular space were found at the coronal half of the root length between first and second molars, between first molar and second premolar, and between first and second premolars; the worst values were found between the four mandibular incisors. The measurements of interradicular spaces were used to depict the average interradicular sites map (Fig 2).

**Table 1:** Distance of the available 3 mm of interradicular space from the CEJ (%).

Maxilla							
	7_6	6_5	5_4	4_3	3_2	2_1	1_1
Mean	92.7	73.5	92.2	90.5	63.7	97.1	54.9
SD	19.8	24.4	15.4	14.2	25.8	7.3	23.2
Mandible							
	7_6	6_5	5_4	4_3	3_2	2_1	1_1
Mean	43.3	50.5	46.9	67.1	71.7	96.5	91.9
SD	34.6	28.7	25.9	22.9	20.5	12	12.4

7_6 = interradicular space between second and first molars; 6_5 = interradicular space between first molar and second premolar; 5_4 = interradicular space between second and first premolars; 4_3 = interradicular space between first premolar and canine; 3_2 = interradicular space between canine and lateral incisor; 2_1 = interradicular space between lateral and central incisors; 1_1 = interradicular space between central incisors.

Concerning crowding, the most crowded segments were the mandibular anterior segment, followed by the maxillary anterior segment (Table 2).

**Table 2:** Crowding measured on digital models (mm).

Maxilla				
	Right posterior	Anterior	Left posterior	Total
Mean	-0.21	-1.84	-0.11	-1.95
SD	0.85	2.54	0.48	3.12
Mandible				
	Right posterior	Anterior	Left posterior	Total
Mean	-0.34	-2.09	-0.34	-2.65
SD	0.78	2.52	1.01	2.9

The Kendall’s tau b test for maxilla revealed a statistically significant correlation (*p*< 0.05) between crowding and the availability of space between the canine and the lateral incisor (Table 3). In the mandible, Kendall’s tau b test revealed a statistically significant (*p*< 0.01) negative correlation between crowding and the availability of space between first and second premolars (Table 3).

**Table 3:** Correlation between crowding and amount of interradicular space.

	7_6	6_5	5_4	4_3	3_2	2_1	1_1
Maxilla	0.02	0.07	0.061	0.083	0.170*	-0.026	0.213
Mandible	-0.112	-0.045	-0.224**	-0.079	-0.004	0.004	0.005

*p< 0.05; **p< 0.01; 7_6 = interradicular space between second and first molars; 6_5 = interradicular space between first molar and second premolar; 5_4 = interradicular space between second and first premolars; 4_3 = interradicular space between first premolar and canine; 3_2 = interradicular space between canine and lateral incisor; 2_1 = interradicular space between lateral and central incisors; 1_1 = interradicular space between central incisors.

## DISCUSSION

Digital panoramic radiograph is a simple, low-cost exam, with a small radiation exposure, with good diagnostic capabilities, and broadly used for screening and initial diagnosis in almost all dental practices.^27^ On the other hand, panoramic radiograph is a kind of tomography imaging where a narrow X-ray beam and a detector rotate simultaneously around the patient’s head with multiple centers of rotation, trying to depict a horseshoe-shaped focal trough, as similar as possible to the shape of the mandible. Objects situated outside the focal trough are reproduced with characteristics distortions, and the degrees of magnifications vary in the horizontal and vertical planes, therefore, is difficult to obtain precise measures. Nevertheless, as for other studies,^11^ ^,^ ^12^ it was decided to use panoramic radiographs for the present investigation due to their low radiation dose and because it is closer to what is routinely done in a dental practice, as the use of other sophisticated exams like CBCT is not routinely recommended for miniscrew placement.^28^

The use of digital models instead of dental casts offers several advantages: analyzing data is easier and faster, there is no need to handle dozens of plaster models and no risk to damage them, data can be directly exported to a database for management, reducing possible errors due to manual transcription; furthermore, casts analysis and measurements made on digital models proved to be as accurate and reproducible as those made on plaster models,^26^ ^,^ ^29^ and some other authors found that digital models offer even greater reproducibility.^30^

The first aim of the present study was to define a map of average interradicular sites to provide the clinician information useful during the very beginning of orthodontic treatment planning.

In this study, the value of 3 mm of bone between two adjacent roots was used as a minimum value of available bone for the placing of a miniscrew of 1.5 mm diameter, taking into account 0.5 mm of bone surrounding the miniscrew and 0.25 mm per side of periodontal width.^11^ ^,^ ^16^ In light of this consideration, in the present sample a miniscrew could have been placed far halfway the length of the roots in most interradicular spaces. Considering that usually the mucogingival line is coronal to the 50% of the root length, this means that in most of the cases is really difficult to place a miniscrew into attached gingiva, at least referring to mean values of gingiva height found in the literature.^31^ This is of great importance, as having the miniscrew head surrounded by attached gingiva is considered one of the main factors for clinical success.^23^ Values smaller than 50% were found in the mandible between second molars and first molars, between first molars and second premolars, and between second and first premolars. In the maxilla, the most favorable sites were between first molar and second premolar, between canine and lateral incisor and between the two central incisors. These results are consistent with the findings of Schnelle et al:^11^ in a study on digital panoramic radiographs, they assessed that at most sites adequate bone for placement was located more than halfway down the root length, and that the only place where was possible to place a miniscrew into attached gingiva was between mandibular second and first molars. Moreover, they found that the interradicular sites with the best amount of bone were between first molar and second premolar, canine and lateral incisor and between the central incisors in the maxilla; in the mandible, the best locations were between second and first molar, first molar and second premolar, canine and lateral incisor. Their results are in accordance with those of the present study.

Furthermore, the authors recorded the same measurements on the post orthodontic treatment panoramic radiographs of the same patients: this way they assessed that, having roots parallel and aligned following orthodontic treatment assures in general a greater number of available interradicular spaces.^11^

The second aim of the present study was to evaluate if a correlation between crowding and availability of interradicular space exists, that could be used as a non-radiological method to estimate the feasibility of miniscrew insertion. Despite the presence of some severely (more than 10 mm) crowded patients, this sample on average exhibited a mild (less than 4 mm) crowding of the arches. With respect to crowding, statistical analysis in the present study revealed that a correlation existed between maxillary canine and lateral incisor, mandibular first and second premolar and the crowding of the respective arch segments, therefore the null hypothesis should be partly rejected. The data for maxillary canine and lateral incisors are in accordance with those of Schnelle et al:^11^ less crowding means more space for miniscrew positioning.

The correlation between mandibular first premolar and second premolar was positive, which means that more crowding results in more space between the roots. This finding can be confusing. Probably, that’s because in most of the cases mandibular premolars were rotated. A rotated tooth occupy a space in the arch greater than its mesio-distal width: therefore, when measuring tooth-arch size discrepancy, an excess of space will come out; on the other hand, when measuring interradicular space on panoramic radiograph, a rotated root has an apparent greater width (since panoramic imaging is a two-dimensional projection and roots are wider in the linguo-buccal direction) and there seems to be less available bone. 

In light of the outcomes of the present study, when planning miniscrew insertion at the very beginning of orthodontic treatment outlining, the clinician should be aware of the average interradicular sites map herein provided, which suggests where most likely miniscrews could be placed, and that in presence of crowding the actual interradicular space available for some areas might be significantly different from average values reported in the literature. An individualized investigation for every single patient, with a radiographic technique different from panoramic images, is still recommended prior to miniscrew insertion.

## CONCLUSIONS

Within the limitations of the present study, mandibular interradicular sites where an amount of 3 mm of bone and in a place covered by attached gingiva are to be most likely found between second and first molars, between first molar and second premolar, and between second and first premolars; in the maxilla, they could be found between first molar and second premolar, between canine and lateral incisor and between the two central incisors.

In addition, some interradicular spaces were found to be influenced by the presence of crowding in the arches. Therefore, in the initial phases of the orthodontic treatment and when miniscrews are needed, the clinician should be aware of these correlations that can alter what is commonly reported in the literature. 
